# Variola

**Published:** 1872-06

**Authors:** A. S. Payne

**Affiliations:** Virginia


THE GEORGIA

### MEDICAL COMPANION:

A MONTHLY ADVISER.

VOL. 2. ATLANTA, GA., JUNE, 1872. No. 6

### PART I.

### Original Communications and Special Selections.

### VARIOLA.

BY A. S. PAYNE, OF VIRGINIA.

[*Continued from May Number.*]

It should be borne in mind that this family of Mr. Elsea's consisted of twelve whites and one colored woman, all of robust, vigorous constitutions, not afraid of manual labor, free livers, that is, on the real substantials of life, consuming daily full rations of pork, fried ham, eggs, etc., etc. The next to the youngest child, a little girl of some eight or nine summers, though robust, was, I found, subject to periodical attacks of phthisis. The mother's distress of mind was great about this child, her only daughter, firmly believing that this delicate child (as she considered it) must "go up" before the terrific assaults of the small-pox, I tried to convince her to the contrary, telling her I believed I could modify its power, etc. With this purpose in view, some three or four days before I expected the appearance of the small-pox eruptions, I placed this child on a restricted diet, (cold rice, milk, etc.) gave her several bilious purgatives, followed by saline cathartics (sulphate magnesia), with an occasional antimonial expectorant, combined with diuretics. This plan succeeded admirably. This child was not compelled to go to bed at all, only a few pustules appearing over her face, arms and body, and those of a smaller size than upon the rest of the patients at this house. This experiment I repeated several times in other families during this epidemic, and always with the same satisfactory results. A robust young colored woman and myself nursed and attended to the general necessities of these twelve small-pox patients. I had been vaccinated in childhood, and again in New York seventeen years before. This young colored woman had a good scar—small, circular, and well pitted—does not know when she was vaccinated. I vaccinated her and myself as soon as I received reliable matter. It appeared to take with both of us, but left no pitted scar. She complained for three days, of headache, pain in her back, and some nausea at the stomach; did not have to go, however, regularly to bed. I would feel well enough in the day, but at night would, for about a week, feel too warm about my face, suffered headache, and great pain in the small of my back. No eruption whatever made its appearance upon either of our persons. I rather attribute my malaise to great fatigue, added to a novel sort of employment, cutting wood, etc., something I had not done since a small boy. I will here remark that this young colored woman exhibited quite a freak of nature. She had (not a cleft of the soft palate) two large, distinct, well developed uvulas. This house was so well quarantined by fear and by my advice, that not a single case occurred emanating from it, although there were twelve cases, eleven of which were of as virulent, confluent form as I ever saw in the "slums" of New York City. On her way home from the protracted meeting, Miss W. called by Mr. Elsea's. I was chopping wood for the colored woman to get supper with. Just as I saw this young lady stepping into the door, I hailed her with, "Don't go in there! They have got the small-pox in that house!" "It is nothing but the chicken-pox; father and mother have it now at home." "Oh well, if you know better than I do, then go on." In a very few moments she came out evidently much alarmed, and asked me to feel her pulse, and to vaccinate her. Putting down my axe, I placed my hand on her wrist, and lo, there was that same peculiar, never to be forgotten, pulse. I said to her, "It is too late for vaccination to do any good; you already have the disease. You had better get across the river as quickly as possible; you will break out with the disease to-morrow." I learned afterwards that she did so break out, and was a corpse in less than ten day's time. After Mr. Elsea's recovery he frankly admitted that he called to see Mr. Wood and his "sick family" on the evening of the day he got so drenched with rain on his return home from Millwood. Mr. E. then contracted the disease from a house in which there were certainly two deaths, and the rest of the cases were no doubt of the confluent type of the disease.

About the first of January following, Mr. J. L. paid a visit on the west side of the Shenandoah River, and brought back with him the germs of variola. His family soon became affected. I did not see this family until one death had occurred, and up to which time it was considered the chicken-pox; therefore no quarantine being established, the disease spread to a considerable extent in that portion of Clarke county popularly known as the "Pine Hills." I suppose, contracted from this house, from first to last, I must have seen as many as a hundred and fifty cases, the majority of which were, however, mild. Occasionally a severe case would present itself in those patients of a vigorous constitution, with a gross habit of body.

By the first of March, 1864, no new case occurring for some length of time before, I began to congratulate myself upon having finally gotten rid of the small-pox. The 10th day of March, 1864, a party of Col. Mosby's command, returning from a raid in the Valley, brought four wounded prisoners to my office in Paris, Fauquier county, for me to extract the bullets and dress their wounds. I noticed that one of the party, a raw Dutchman, although more slightly wounded than the rest (only a slight flesh wound) nevertheless halloed louder, and made a greater fuss generally, than all the rest combined. From some cause the Dutchman was left at Mr. A.'s hotel, at the lower end of the village, at the same time the men seriously wounded were carried on to Richmond. As was my habit, I had prescribed cold water dressing for all their wounds after the abstraction of the balls. Riding by the hotel on the fourth day, after the abstraction of the ball, the aforesaid Dutchman requested me to stop and examine his wound, saying it pained him very much, and he felt sick besides. An occasional pimple appeared around the edges of the wound, and there was evidence of slight inflammatory action going on. The suspicious character of these pimples caused me to examine his face, body, and limbs: scattered over each of these parts I also detected a few straggling papulæ. I feel his pulse and found that peculiar pulse, which I contend is pathognomonic of variola. This morbid condition of his system explained the suffering he experienced by so slight an operation. Had I felt his pulse at the time of the operation, I should no doubt have detected the incipient stage of variola, under which he was laboring at that time. He was ignorant as to how long since he had been vaccinated. It has evidently been a long time since, probably twenty-five years, for the scar and pitts are considerably dimmed by the lapse of time. Yet we find the disease greatly modified by the ancient vaccination. The hotel at which this Dutchman was domiciled, in number of persons, regular inmates, reached from fifteen to twenty, with a very large number of citizens and soldiers hourly passing to and fro. In some six or eight days I was again summoned to this hotel. Found the Dutchman well and hearty, but three cases of variola in the house, the patients respectively having been vaccinated from seventeen to twenty years before. At a house just opposite, from which there was daily communication, I found three cases of varioloid, and one case of confluent small-pox. This case was a negro child about two years of age, and was from home when I vaccinated the colored portion of this family the fall before. These cases had been vaccinated upwards of twenty years before, and consisted of five whites and seven blacks. At a house about one hundred yards distant, in a family of three whites and seven blacks, I found one case of confluent small-pox. This case, a young negro man, had never been vaccinated at all. So far as I know, these were all the cases attributable to the visit of the Dutchman to this village. And here, before I forget it, I wish to record one remarkable fact.

Miss W., who we have seen attended the protracted meeting, and then returned home (by Mr. Elsea's) to die with the small-pox, remained two nights at her relative's, Mr. W.'s, where there were fourteen susceptible persons, and one night at Mr. F.'s, where there were sixteen susceptible or unvaccinated persons. On the forenoon of November 3, 1864, I vaccinated a large per cent. of the inhabitants of the village of Paris. In the afternoon I rode over and vaccinated the whole of Mr. W.'s family, white and black, save his eldest daughter; she had such a large scar (as large as a quarter of a dollar, with one deep pit in the centre) that she declined to be vaccinated, although I strongly urged her to the contrary. On the next day I was called to see Mr. F.'s family, they supposing I had given them the small-pox I found them all, white and black, in bed with angina, headache, pain in the back, and nausea, the pulse closely resembling that belonging to variola. From curiosity, I was led to return by Mr. W.'s, and found his family the duplicate of Mr. F.'s. These symptoms all passed off in the course of three days without any appearance of eruption. The persons I vaccinated in Paris in the morning, with the same vaccine virus, none of them complained at all until the seventh or eighth day, then only of slight indisposition or slight nausea, many never complaining at all. What was the cause of this? I incline to the opinion that the inhabitants of these two exposed houses had in their persons already the germs of variola, partially developed, and the struggle between the vaccine virus (the antidote of the disease) and the disease for the mastery, was of such a fierce and of such a sharp character as to make the person in whose system the grand fight occurred absolutely sick. What do you think of this hypothesis, gentlemen? I had nearly forgotten to mention that on the 7th of November, Mr. F.'s eldest daughter broke out with variola in the confluent type of the disease. I thought she came nearer dying with the disease than any of my cases.

On the 10th of July a colored man, a former servant of and living with Mr. O., came to Mr. O.'s from his wife's house near Millwood, and in a few days broke out with confluent small-pox. Mr. O.'s family consisted of Mr. and Mrs. O., Mrs. S., a married daughter, two young children, Miss O., two black men, black women, and three or fo r children. Mrs. O., Miss O., the women and children, were vaccinated by me the fall before. Mr. O. had been vaccinated probably thirty years before: the negro men, if they had ever been at all, which I do not now remember, was a number of years before. The other negro man first took the disease. Mr. O., who was awfully afraid of the disease, passing by the window after he thought all danger had vanished, asked the servant if he wished anything to eat. He took the disease thus easily. The two negro men, Mr. O., and Mrs. S.'s two children, had the disease in its confluent form, Mrs. S. herself in a modified form, whilst Mrs. O., Miss O., the negro woman and the three or four children, vaccinated by me the fall before, showed no symptoms of the disease. Several cases occurred among the blacks, contracted at this house, all of which were of the confluent form.

My treatment of small-pox may be said to be emphatically the cold plan of treatment. The room should be kept cool, clean and dark. The diet cold, light, and anti-phlogistic. The patient should be allowod iced jellies, iced lemonade, and iced rice, milk, *ad libitum.* Where a tonic is desirable, the nitro muriatic acid, sweetened with loaf sugar and dissolved in ice water, will be found to be beneficial. The acids seem to be particularly called for in variola. I would, in all cases of confluent small-pox, commence by giving a purgative dose or two of sub. mu. hydrargyrum, alone or combined with compound extract colycinth, at bed time, to be followed next morning by a cooling saline cathartic, such as sulphate of magnesia or Sedlitz powder. Bilious purgatives, low diet, and cooling saline cathartics, have a modifying influence second only to vaccination. Alcoholic stimulants I cannot recommend in variola. If there is a purplish appearance about the pustules, or a manifest depression of the system after the secondary fever has passed, I would (instead of cordials, wine, etc.,) recommend cold tea, combined with opiates to allay nervous irritation, and the free use of beef tea, iron, and the mineral acids. I will only say in reference to vaccination, that it is a neat little operation, often done improperly by unprofessional persons, and its beneficial effects defeated by drawing too much blood, and by iritating the arm too much inflammation is set up, and the vaccine virus destroyed thereby. If your vaccine matter is good, your operation will rarely fail, if before you vaccinate, you make the patient take a brisk walk in the open air; if a child make the nurse take it out and bring on a glow. The operation mostly fails from a torpid, sluggish state of the cutaneous circulation. Rouse the pulse by exercise to 100, it will then be rapidly carried into the system, and if the matter is at all good, I will insure it rarely to fail to take, even in the pale, flabby, yellow, cadaverous-looking skin.

I saw cases of variola from 1840 to 1846 almost daily, in 1863 I treated about two hundred cases, and I feel myself justified, from my experience, in drawing the following conclusions:

1. That to afford certain protection the scar should present the following appearances: the cicatrix should present a peculiar dotted appearance; should be smooth, glossy, and shining; pearly white in color; circular or ovate in form, with slight depression below the surface of the surrounding skin; with a pellicle-like thinness of the new cuticle covering the cicatrix; cicatrix should not be over half an inch in length and less in breadth.

2. That in some constitutions, where vaccination has been properly performed, and the vaccine virus fresh, it never wears out; in other constitutions it wears out entirely after twenty years, and loses much of its influence after fifteen years.

3. That I would recommend every one to be vaccinated every seven years; that I have never seen a case of either variola or small-pox occurring in a person vaccinated within as short a time as seven years.

4. That it is absurd to assert that vaccination affords no protection from small-pox or from varioloid. I believe if vaccinated only two days before the eruption, it will modify the severity of the attack.

5. That small-pox is very amenable to proper treatment. That it surely is greatly modified by restrictive diet and purgative medicines.

6. That the chief terror of this disease lies in its more contagious character, combined with the horrible transformation, and the awful effluvia arising from the patient's body during the period of incubation to that of maturation.

7. That in my opinion a "hard and frequent pulse" does not necessarily describe the pulse of any of the varieties of small-pox; it is *sui generis*. That the pain in the loins is a surer index of the severity of the attack than vomiting or any other symptom.

8. That the variola nigra of Sydenham, the bloody small-pox of Mead is the same disease as confluent small-pox in old, worn-out, broken-down constitutions.

9. That if you vaccinate persons who have already taken the germs of the disease in their systems, they become very rapidly under the influence of the vaccine virus.

I do not wish to be understood as insinuating that any of my professional brethren mistook small-pox for chicken-pox. So far as I know, I was the only practicing physician in a radius of twenty miles; all were with the army or had refugeed. Under more favorable circumstances, I would voluntarily have crossed the Shennandoah, and changed the plan of treatment, but blue coats were continually prowling around on that side, and only occasionally coming on my side, or Mosby's Confederacy. It was extremely fashionable to pick a man up and send him to Camp Chase, Fort Delaware or Fort Warren, and as a doctor was about then a sort of "rara avis," I did not know but that as a matter of personal distinction to me they might send your humble servant to Fort Leavenworth, or the Sandwich Islands.

